# Drug-resistant tuberculosis: integrative review of nursing care in primary health care

**DOI:** 10.1590/0034-7167-2023-0097

**Published:** 2024-07-19

**Authors:** Sibele Naiara Ferreira Germano, Alacoque Lorenzini Erdmann, Camila Freire Albuquerque, Lúcia Nazareth Amante, Darlisom Sousa Ferreira, Marlucia da Silva Garrido

**Affiliations:** I Universidade Federal de Santa Catarina. Florianópolis, Santa Catarina, Brazil; II Universidade do Estado do Amazonas. Manaus, Amazonas, Brazil; III Universidade Federal do Amazonas. Manaus, Amazonas, Brazil

**Keywords:** Tuberculosis, Tuberculosis, Multidrug-Resistant, Nurses, Nursing Care, Primary Health Care, Tuberculosis, Tuberculosis Resistente a Múltiples Medicamentos, Enfermeras y Enfermeros, Atención de Enfermería, Atención Primaria de Salud

## Abstract

**Objectives:**

to identify, in the scientific literature, the care that should be provided to individuals with drug-resistant tuberculosis by nurses in primary health care.

**Methods:**

integrative review, using the Preferred Reporting Items for Systematic Reviews and Meta-Analyses study selection flowchart. Data collection was conducted in November 2022, across ten databases.

**Results:**

six studies emphasized that nurses should perform directly observed treatment; two highlighted the importance of integrated care management between tuberculosis and human immunodeficiency virus; two demonstrated comprehensive nursing consultation using the nursing process; one emphasized person-centered care, with discharge planning and improved hospital communication with primary health care services.

**Final Considerations:**

the care that nurses should provide to individuals with drug-resistant tuberculosis in primary health care, for care resolution, is evidence-based.

## RESUMEN

**Objetivos:**

identificar, en la literatura científica, los cuidados que deben brindarse a las personas con tuberculosis farmacorresistente por parte de los enfermeros en la atención primaria de salud.

**Métodos:**

revisión integradora, utilizando el flujo de selección de estudios del *Preferred Reporting Items for Systematic Reviews and Meta-Analyses*. La recolección de datos se realizó en noviembre de 2022, en diez bases de datos.

**Resultados:**

seis estudios enfatizaron que el enfermero debe llevar a cabo el tratamiento directamente observado; dos resaltaron la importancia de la gestión del cuidado integrado entre la tuberculosis y el virus de la inmunodeficiencia humana; dos evidenciaron la consulta de enfermería comprensiva utilizando el proceso de enfermería; uno destacó los cuidados centrados en la persona, con planificación de alta y una mejor comunicación hospitalaria con los servicios de atención primaria de salud.

**Conclusiones:**

los cuidados que deben brindarse por parte de los enfermeros a las personas con tuberculosis farmacorresistente en la atención primaria de salud, para la resolutividad del cuidado, están basados en evidencia científica.

## INTRODUCTION

It was believed that Tuberculosis (TB) could be eradicated by 2035 through the implementation of control strategies, free treatment, and advancements in early diagnostic methods. However, the emergence of the COVID-19 pandemic has hindered progress in eliminating TB as a global public health issue, with a 25% reduction in diagnoses and a 26% increase in mortality from the disease^(1-2)^. This challenge is compounded by the escalation of its severe form, Drug-Resistant Tuberculosis (DR-TB), which was recently estimated to have 450,000 new cases by the World Health Organization (WHO)^(1)^.

TB is categorized as DR-TB when Mycobacterium tuberculosis develops resistance to one or more anti-tuberculosis drugs. There are five manifestations of this disease: TB resistant to a single medication (monoresistance); resistance to two or more medications (polyresistance), excluding the rifampicin and isoniazid combination; the resistant one detected by the rapid molecular test for tuberculosis (resistance to rifampicin); resistance to at least rifampicin and isoniazid (multidrug resistance); and resistance to rifampicin and isoniazid, coupled with resistance to any fluoroquinolone and at least one of the drugs bedaquiline and linezolid (extensive resistance)^(2-3)^.

The treatment regimen and duration for DR-TB depend on its form, varying from 18 to 24 months or longer, taking into account clinical, radiological, and bacteriological developments. The complexity of this treatment is heightened by the necessity for second-line medications, whether oral, injectable, or both, which can induce more severe side effects. Recently, new fully oral treatment regimens for DR-TB have been introduced, potentially reducing treatment duration by up to 6 months compared to previous protocols^(4)^.

Given this evolving landscape, WHO advocates for expanding access to fully oral treatments, underscoring the imperative to bolster nursing care in primary health care (PHC). PHC, operating at the grassroots level and intimately connected to individuals, families, and communities, is crucial for providing support, counseling, and monitoring adverse events^(1,4)^. It is crucial to note that nursing care for individuals with DR-TB must be comprehensive across all three levels of care: primary, secondary, and tertiary, with PHC serving as the nexus for coordination. However, it is essential that PHC be staffed with qualified professionals, including advanced practice nurses, who play a pivotal role in enhancing accessibility and delivering superior management for individuals afflicted with this disease at this level of care^(5-6)^.

In PHC settings, the rate of loss to follow-up for individuals with DR-TB who receive nursing care is lower than those solely treated in hospitals, and the treatment success rate for individuals receiving care for this disease in PHC is higher compared to those solely treated in hospitals^(7-8)^.

The Ministry of Health (MoH) in Brazil, along with other countries, recognizes the role of advanced practice nurses in caring for individuals with DR-TB in PHC. In 2022, the MoH launched the TB treatment protocol for nurses, which includes recommendations related to DR-TB care in PHC. This protocol encompasses nursing consultations, planning, management, coordination, and evaluation of actions conducted by qualified nurses with clinical competence^(6,9-10)^.

With the increasing prevalence of DR-TB, the introduction of new oral treatment regimens, and the necessity to strengthen care in PHC, this study is justified by the understanding that reviewing the literature on the care provided by nurses to individuals with DR-TB in PHC will furnish scientific evidence regarding the care that should be administered by these professionals when dealing with this serious disease at the local level, involving individuals, families, and communities. This will improve access and adherence to the correct treatment until the cure of this disease, which impacts global public health, is attained^(4-6)^.

## OBJECTIVES

To identify, in the scientific literature, the care that should be provided to individuals with drug-resistant tuberculosis by nurses in primary health care.

## METHODS

A descriptive integrative review study, which synthesizes results of scientific research on a specific topic in a systematic and orderly manner, conducted in six stages: identification of the theme and definition of the guiding research question; establishment of criteria for inclusion and exclusion of studies; categorization of studies; critical evaluation of included studies; interpretation of results; description of the synthesis of scientific knowledge of the investigated theme, contributing to evidence-based practices^(11)^.

The first stage, defining the research question, arose from the PICo strategy, which stands for: P - Participants, I - Phenomenon of Interest, and Co - Context^(12)^. In this study, the following components were assigned: P - nurses, I - care provided to individuals with drug-resistant tuberculosis; Co - primary health care, resulting in the following guiding question: What care should nurses in primary health care provide to individuals with drug-resistant tuberculosis?

The inclusion criteria defined for the search of studies in the databases were: qualitative and/or quantitative studies; randomized studies; addressing the care to be provided by nurses to individuals with drug-resistant tuberculosis; using the criterion of advanced nursing practice in caring for individuals with drug-resistant tuberculosis in primary care; and care provided to individuals with drug-resistant tuberculosis by the multiprofessional team in primary care, including the nursing professional; in the national and international context; in Portuguese, English, and Spanish languages, published in full in the last five years (2018 to 2022). Exclusion criteria were literature reviews, reflective studies, experience reports, and those not adhering to the guiding question of the research.

Data collection was conducted in November 2022, in the following databases: Cumulative Index to Nursing and Allied Health Literature (CINAHL), Latin American Literature in Health Sciences (LILACS), Nursing Database (BDENF), National Library of Medicine (PubMed)/Medical Literature Analysis and Retrieval System Online (MEDLINE), Excerpta Medical Database (EMBASE), Cochrane Library, Scopus, Web of Science, Scientific Electronic Library Online (SciELO). Search strategies were adapted, with the assistance of a librarian, testing more than one strategy before arriving at the final definition, using terminologies defined by the Medical Subject Headings (MeSH) and Health Sciences Descriptors (DeCS) as per the databases, with the use of boolean operators OR and AND, described in Chart 1.

**Chart 1:** Databases with search strategies using Medical Subject Headings and Health Sciences Descriptors, Florianópolis, Santa Catarina, Brazil, 2022

Database	Search Strategies
Cumulative Index to Nursing and Allied Health Literature National Library of Medicine/ Medical Literature Analysis and Retrieval System Online Excerpta Medical Database Cochrane Library Scopus Web of Science	(Tuberculosis OR “Koch Disease” OR “Koch’s Disease” OR “Kochs Disease” OR “Mycobacterium tuberculosis Infection” OR “Mycobacterium tuberculosis Infections” OR *Tuberculoses* OR “Tuberculosis, Multidrug-Resistant” OR “Drug-Resistant Tuberculosis” OR “MDR Tuberculosis” OR “Multi-Drug Resistant Tuberculosis” OR “Multidrug-Resistant Tuberculosis” OR “Tuberculosis, Drug Resistant”) AND (Nurses OR Nurse OR “Nursing Personnel” OR “Registered Nurse” OR “Registered Nurses” OR “Nursing Care” OR “Nursing Care Management”) AND (“Primary Health Care” OR “Health Care, Primary” OR “Primary Care” OR “Primary Healthcare”)
*Literatura Latino-Americana de Ciências da Saúde* *Banco de Dados em Enfermagem*	(*Tuberculose* OR Tb OR “*Infecção por* Mycobacterium tuberculosis” OR “*Pneumologia Sanitária*” OR Tb OR Tuberculosis OR “*Infección por* Mycobacterium tuberculosis”) AND (“*Enfermeiras e Enfermeiros*” OR *Enfermeira* OR “*Enfermeira e Enfermeiro*” OR “*Enfermeira Registrada*” OR *Enfermeiras* OR “*Enfermeiras Registradas*” OR “*Enfermeiro e Enfermeira*” OR “*Enfermeiro Registrado*” OR “*Enfermeiros e Enfermeiras*” OR “*Enfermeiros Registrados*” OR “*Enfermeras y Enfermeros*” OR *Enfermera* OR “*Enfermera Registrada*” OR “*Enfermera y Enfermero*” OR *Enfermeras* OR “*Enfermeras Registradas*” OR “*Enfermero Registrado*” OR “*Enfermero y Enfermera*” OR “*Enfermeros Registrados*” OR “*Enfermeros y Enfermeras*” OR “*Cuidados de Enfermagem*” OR “*Assistência de Enfermagem*” OR “*Atendimento de Enfermagem*” OR “*Cuidado de Enfermagem*” OR “*Gestão da Assistência de Enfermagem*” OR “*Sistematização da Assistência de Enfermagem*” OR “*Atención de Enfermería*” OR “*Cuidado de Enfermería*” OR “*Cuidados de Enfermería*”)
Scientific Electronic Library Online	(“*Tuberculose Resistente a Múltiplos Medicamentos*” OR “*Tuberculosis Resistente a Múltiples Medicamentos*”) AND (*Enfermeiros* OR *Enfermeros* OR “*Cuidados de enfermagem*” OR “*Atención de Enfermería*” OR *Enfermagem*)

Access to the databases occurred through the Periodicals Portal of the Coordination for the Improvement of Higher Education Personnel in an area with recognized Internet Protocol (IP) from the Federal University of Santa Catarina. The search, selection, and inclusion of studies were carried out independently by two reviewers using the Preferred Reporting Items for Systematic Reviews and Meta-Analyses (PRISMA)^(13)^ study selection flowchart. In cases of disagreement, consensus was sought with the support of a third reviewer, who provided input for the decision regarding inclusion. The lists of studies selected in this phase were compared, and the agreement of the analysis was verified using the Kappa test^(14)^.

The bibliographic manager EndNote Basic^®(15)^ web version assisted in organizing the searches by excluding duplicate articles. The publications found were exported using the Mendeley^®^ Data Manager^(16)^. These data were subsequently grouped and exported into an RIS file using MS Office Excel^®^ software to further process data extraction.

The extracted data were organized and synthesized using a specific form constructed by the researchers, containing the following information: author(s) identification, title, journal, country, study language, and year of publication; objective; study methodology; care that should be provided to individuals with DR-TB in PHC; main results; conclusions; methodological limitations (risk of bias), inconsistency, indirect evidence, imprecision, publication bias, and level of evidence of the studies according to the Grading of Recommendations Assessment, Development, and Evaluation (GRADE)^(17)^.

**Figure 1:**
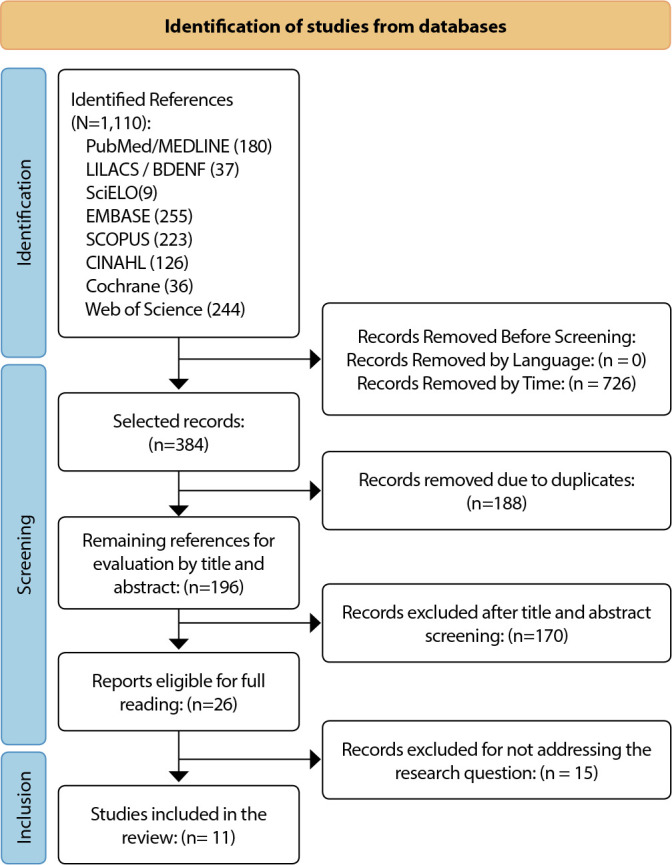
Study selection flowchart adapted from the Preferred Reporting Items for Systematic Reviews and Meta-Analyses 2020^(13)^, Florianópolis, Santa Catarina, Brazil, 2022

The search strategies retrieved 1,110 articles from the databases. Of these, 914 were excluded after applying filters according to pre-established criteria, resulting in 196 for title and abstract reading, of which 170 were excluded. Twenty-six studies were eligible for full reading, resulting in 11 studies that answered the research question of this review, as shown in the flowchart in Figure 1.

## RESULTS

The studies included in the description and synthesis of this review^(18-28)^ provide insights into the care that nurses in PHC should provide to individuals with DR-TB, highlighting the importance of these professionals in promoting health, preventing, and adequately treating the disease. These care practices are grounded in protocols and guidelines stemming from comprehensive public health policies that consider the individual, family, and community within their life context and within a healthcare system with reference and counter-reference, articulating the three levels of care primary, secondary, and tertiary for comprehensive and effective care, as presented in Chart 2.

**Chart 2:** Identification of articles published between 2018-2022 with a description of nursing care for individuals with Drug-Resistant Tuberculosis in Primary Health Care, Florianópolis, Santa Catarina, Brazil, 2022

Study Title Methodological	Year Country	Design	Main Results/Conclusions of Nursing Care	Level of Evidence GRADE
An intervention to optimise the delivery of integrated tuberculosis and HIV services at primary care clinics: results of the MERGE cluster randomised trial^(18)^	2018 South Africa	Randomized cluster trial	Integrated care management for TB/HIV with early diagnosis and guideline-based treatment, including contact tracing.	High 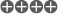
Nurses’ Knowledge of Tuberculosis, HIV, and Integrated HIV/TB Care Policies in Rural Western Cape, South Africa^(19)^	2018 South Africa	Qualitative and quantitative	Integrated TB/HIV care management following extensive training of professionals in TB guidelines and its severe form, DR-TB.	High 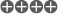
Continuity of care for TB patients at a South African hospital: A qualitative participatory study of the experiences of hospital staff^(20)^	2019 South Africa	Action research	Person-centered care, with discharge planning and improved hospital communication with PHC services.	Low 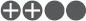
*Percepções de enfermeiros sobre gestão do cuidado e seus fatores intervenientes para o controle da tuberculose^(21)^*	2022 Brazil	Qualitative	Nursing consultation, monitoring of confirmed cases with control bacilloscopy; Directly Observed Treatment (DOT); treatment of Latent Tuberculosis Infection (LTBI); and health education.	Moderate 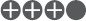
*Consulta de enfermagem a pessoas com TB: proposta de instrumento^(22)^*	2020 Brazil	Action research	Nursing consultation for individuals with TB and DR-TB using the Nursing Process, encompassing all stages, promoting individualized and appropriate care for each person.	Moderate 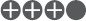
*Representations on adherence to the treatment of Multidrug-Resistant Tuberculosis^(23)^*	2018 Brazil	Qualitative	Welcoming, attentive listening, clarification of doubts, and establishing rapport. Implementation of DOT to ensure treatment adherence and monitor medication side effects; providing health education to patients and their families.	Low 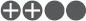
*Coordination and list of services for the management of tuberculosis: perspective of health professionals^(24)^*	2022 Brazil	Quantitative	DOT, including home visits for monthly monitoring and supervision of patients and their contacts; conducting rapid HIV testing for all TB patients; ensuring smooth referral and counter-referral processes with TB, DR-TB, and HIV reference services.	Low 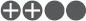
Nurses’ discourses on actions and strategies of care for the elderly with tuberculosis^(25)^	2022 Brazil	Qualitative	Comprehensive care encompasses active case finding, incentives, home visits, health education, and examinations.	Very Low 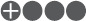
*Percepção de enfermeiros: enfoque na família e orientação para a comunidade nas ações de TB^(26)^*	2020 Brazil	Quantitative	Engagement of TB patients in care plan development and planning process, while respecting and fostering autonomy in carrying out DOT, strengthening relationships, implementing promotional and preventive measures, screening for respiratory symptoms, and providing health education.	Very Low 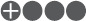
*Passos e descompassos no processo de cuidado aos portadores de tuberculose na atenção primária^(27)^*	2020 Brazil	Qualitative	Care delivery aligned with the policies and strategies of the World Health Organization: early diagnosis through active case finding, HIV testing, DOT, and ongoing education to promote evidence-based practice.	Low 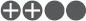
Provider perspectives of the introduction and implementation of care for drug-resistant tuberculosis patients in districtlevel facilities in South Africa: a qualitative study^(28)^	2020 South Africa	Qualitative	Decentralized care involving DOT, monitoring of side effects, and adherence through monthly sputum collection and routine testing.	Moderate 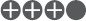

*TB - Tuberculosis; DR-TB - Drug-Resistant Tuberculosis; HIV - Human Immunodeficiency Virus; DOT - Directly Observed Treatment; LTBI - Latent Tuberculosis Infection; SR - Symptomatic Respiratory.*

Regarding nursing care provided by PHC, six studies^(21,24,26-28)^ emphasize Directly Observed Treatment (DOT), two^(18-19)^ highlight the importance of integrated TB/HIV care management, two^(21-22)^ demonstrate comprehensive nursing consultation using the Nursing Process (NP), and one^(18)^ emphasizes person-centered care with discharge planning and improved hospital communication with PHC services.

In addition to these care practices, the studies^(18-28)^ mention other care aspects, also found in WHO policies and strategies, such as welcoming, attentive listening, attention, clarification of doubts, establishing rapport, early diagnosis, home visits with active case finding for Symptomatic Respiratory (SR), rapid HIV testing for all TB patients with referral and counter-referral flow with TB, DR-TB, and HIV reference services, treatment of LTBI, continuous health education, always encouraging evidence-based practice.

When assessing the characteristics of the listed studies and their levels of evidence, seven studies^(21-27)^ were conducted in Brazil and four^(18-20,28)^ in South Africa. Five of these studies^(21,23,25,27-28)^ had a qualitative methodological design, two^(22,24)^ quantitative, two action research^(18,20)^, one^(19)^ qualitative and quantitative, and one^(18)^ cluster randomized.

In evaluating the factors that decrease confidence in the results (study biases, inconsistency, imprecision, indirect evidence, and publication bias) and factors that increase confidence (large effect size, dose-response gradient, conservative biases of each study following the GRADE system)^(17)^, two articles^(18-19)^ included the necessary items to be considered of high methodological quality, and nine^(20-28)^ failed to achieve this level of evidence due to the absence or inadequacy of methodological description of necessary items.

## DISCUSSION

Given the importance of proper TB management for DR-TB control, it is essential to consider that individuals previously treated for TB are more likely to develop DR-TB, either due to inadequate treatment regimens or non-adherence to therapy^(2-3)^. Furthermore, individuals with DR-TB serve as a source of dissemination of resistant strains to other individuals who may primarily acquire this severe form of the disease^(10)^.

In this context, and considering the need for more specific care for individuals with DR-TB, we highlight in two sections the nursing care that should be provided to these individuals in PHC, as emerged from the analyzed studies.

### Continuity of nursing care for individuals with drug-resistant tuberculosis in primary health care

Studies conducted with individuals with DR-TB, utilizing continuity of care between the hospital, referral center, and PHC, demonstrated that this approach was effective for treating these individuals, especially when advanced practice nurses are involved in primary level care. This type of care, close to the sick individual, shows increased access to health services, better disease management, and higher user satisfaction^(23,28)^. These results were observed not only in studies conducted in Brazil but also in studies conducted in South Africa and Peru, where care in clinics near the residences of individuals with DR-TB or home care were effective alternatives to hospitalization throughout treatment^(29-30)^.

Nursing care for individuals with DR-TB should be provided with continuity across all three levels of care (primary, secondary, and tertiary), aiming for comprehensive care and constant surveillance of DR-TB^(4,10,23,28)^. Studies show that PHC nurses should conduct nursing consultations for individuals with DR-TB, request complementary exams, perform DOT, provide guidance on medication side effects according to clinical protocols and therapeutic guidelines, and supervise welcoming with qualified listening and risk classification. It is also the nurse’s role to develop care plans, coordinate and evaluate team actions, including nursing technicians/assistants and community health agents (CHAs), guiding the search for respiratory symptoms in the area (SR)^(6,10,23,28)^.

All nursing care should follow the guidelines of the Programmatic Management of Drug-Resistant Tuberculosis (PMDT), formulated by the WHO. Several countries, such as South Africa, Peru, and Brazil, have specific clinical protocols and therapeutic guidelines for PHC nurses, strengthening this level of care by describing the care that nurses should provide to individuals with DR-TB, including clinical assessment through physical examination during nursing consultations, implementation of DOT with assessment of contacts for latent TB infection (LTBI)^(6,29-30)^.

The need for nursing care for individuals with DR-TB in PHC is evident. A study conducted at a reference hospital for severe TB cases showed failures in the care of sick individuals who had only their treatment carried out by tertiary care (hospital). Nurses expressed concern about the lack of DOT implementation, the lack of follow-up care for individuals with DR-TB, and the follow-up of family members, which should be carried out at this level of care, without proper referral and counter-referral and without the involvement of the PHC health team in the discharge plan and continuity of care^(20)^.

Studies indicate that the lack of continuity of care from specialized to PHC results in deficiencies in comprehensive care, as it overlooks the health needs of individuals with DR-TB, their families, and communities, thereby excluding them from discharge planning. This leads to a disruption in PHC-sensitive care from the central to the local level, leaving PHC nurses unable to perform DOT, education, and counseling for individuals with the disease and their families during nursing consultations. This impairs care and contributes to increased treatment abandonment and disease spread^(20,23)^.

Similar findings emerged in studies conducted in countries with a high TB burden, where effective integration of care between hospitals and TB services in PHC was lacking. Strategies were necessary to integrate care provided in general hospitals and PHC services into the National TB Control Program and its severe form, DR-TB. The importance of coordination structures between levels is underscored, with PHC serving as a critical link in organizing care between different levels, given its proximity to the people. Professionals should focus on disease prevention and early detection, referring individuals to specialized care as needed, and facilitating information sharing^(31-33)^.

However, one study emphasized the importance of individuals with DR-TB initiating treatment in DR-TB reference units and then transitioning to PHC. Here, nurses are responsible for administering daily injections, conducting DOT, and monitoring sputum examination. These individuals should return to the reference unit monthly or bimonthly for treatment review based on the results of monthly sputum examination at PHC^(28)^.

It is evident from several studies^(7-9,20,23,28)^ that PHC-based care for individuals with DR-TB is a successful approach adopted in some countries. This model advocates for outpatient treatment, with nurses overseeing care, ensuring favorable conditions, and reducing hospitalization duration for those in need. By sharing responsibility for care and providing appropriate treatment across all three levels of care, with decentralization from the central to the district and local levels, closer nursing care tailored to the individual can improve treatment adherence and cost-effectiveness.

### Nursing care centered on the individual with drug-resistant tuberculosis, family, and community

Given the increase in DR-TB cases, the necessity for person-centered nursing care to control this disease is paramount. PHC nurses play a critical role from actively identifying symptomatic respiratory cases to diagnosing, implementing treatment, and monitoring. They administer DOT, monitor confirmed cases with follow-up sputum smears until the end of treatment, and track defaulters daily, employing strategies such as Home Visits (HV) and health education to prevent abandonment^(21-22,34)^.

Studies confirm that home visits and health education activities enhance contact tracing, bring families closer to healthcare professionals, and reduce disease stigma^(14,18,20-23)^. These activities are crucial for TB management, influencing the adoption of relevant therapeutic measures like DOT for medication adherence and continued follow-up in PHC^(19,26)^.

DOT is a care strategy introduced by the WHO to bolster TB response capacity. Its primary goals focus on treatment adherence through supervised medication intake and social support, preventing the emergence of drug-resistant strains, reducing abandonment rates, and increasing the likelihood of disease cure^(23,27)^.

PHC nurses, in delivering person-centered care for DR-TB, also engage in contact tracing, treatment of latent TB infection (LTBI) when indicated, completion of surveillance instruments recommended by the National Tuberculosis Control Program (NTCP), and health education activities, emphasizing health promotion and disease prevention^(21-22,24,34)^.

Studies confirm that nurses, when performing Nursing Care Management (NCM), deliver comprehensive and effective care after receiving extensive ongoing health education (OHE) on TB and DR-TB^(19-20)^. This underscores the necessity for ongoing nurse education on DR-TB diagnosis, treatment, and monitoring, supported by a system for successful treatment, and adoption of policies, protocols, and electronic information systems for recording, reporting, and information sharing between hospitals and PHC services.

Person-centered nursing care for DR-TB in PHC currently mirrors a practice that requires strengthening through OHE. Inequalities persist among professionals regarding educational levels in Brazil, necessitating greater involvement of these individuals in attitudes and decision-making in PHC to prevent this disease and promote health, implementing ideal practices rather than the status quo, always encouraging evidence-based practice^(27)^.

Studies show that to develop person-centered care, comprehensive Nursing Care Management (NCM) must be performed, where nurses use management tools such as health indicators, material and human resource planning, safety standards for care, and decision-making processes^(19-21)^. To achieve this, direct care provided to the public should encompass techniques, technologies, procedures, and actions for prevention, promotion, and health education^(21,35)^.

It is essential that professionals engaged in NCM for individuals with DR-TB are prepared for person-centered care in all its dimensions, understanding their role and being equipped to support these users, demonstrating that they can and should act in favor of their health^(21,35)^.

Therefore, nursing care should be focused on the individual who is ill, strengthening democracy and adherence to the correct treatment, involving them in the development of the care plan and planning, with respect and encouragement for autonomy in carrying out DOT. This involves the participation of the population in discussions on the subject and the utilization of community resources by professionals to support adherence to this method^(26)^.

The TB care protocol emphasizes that to ensure comprehensive person-centered nursing care, nurses must conduct nursing consultations comprehensively, utilizing the steps of the Nursing Process: assessment, diagnosis, planning, implementation, and evaluation, promoting individualized and appropriate care for each individual, supported by theoretical frameworks^(21-22,35)^.

When initiating the assessment stage of individuals with DR-TB, some aspects must be considered in the physical examination, including investigating the signs and symptoms of the disease, how long they have been present, contact with individuals with tuberculosis, tests performed during treatment, professional follow-up, nursing care, perceptions, and concerns regarding the disease, and conducting follow-up sputum smears^(23)^.

During the physical examination of these individuals with DR-TB, the nurse should be attentive to tachycardia, tachypnea or exertional dyspnea, increased respiratory rate, decreased or absent bilateral or unilateral breath sounds, post-tussive crackles, asymmetry in respiratory excursion (in cases of pleural effusion), dullness to percussion, and decreased tactile fremitus (in cases of pleural fluid). Additionally, observe fine crackles after coughing, decreased or amphoric breath sounds. Chest pain exacerbated by recurrent coughing may also be present. Associated conditions such as diabetes and HIV should be investigated^(21-22,36)^.

One study^(18)^ conducted in 18 PHC clinics demonstrated that individuals with TB need to be immediately investigated for their main coinfection, HIV, to reduce morbidity and mortality from TB/HIV coinfection, with counseling and rapid HIV testing for all individuals with TB, intensifying active case finding for TB in HIV cases and vice versa, preventive therapy for TB infection control, and early initiation of Antiretroviral Therapy (ART).

The Brazilian Ministry of Health, in line with the WHO, recommends that every individual diagnosed with TB should be tested for HIV, as early diagnosis has a significant impact on the clinical course of both diseases. Positive cases should be referred to the Specialized Care Service (SCS) or drug dispensing units to continue TB treatment and initiate HIV treatment, always ensuring referral and counter-referral within the Health Care Network^(24)^. Therefore, it is evident that every individual with DR-TB should receive comprehensive care according to their needs and comorbidities, by a multidisciplinary team in which the PHC nurse acts as a member as well as a care manager and coordinator^(18-29,35)^.

### Study limitations

The limitations of this study predominantly involve the qualitative approach to data. Although this method encompasses significant and clinically relevant samples, its qualitative nature does not permit the establishment of cause-and-effect relationships. This is because the level of involvement of the sick individual in their care may vary across different scenarios and groups, making it challenging to generalize the results. Another limitation is the failure to explore grey literature, which could offer additional insights and a more comprehensive understanding of the topic. Additionally, excluding articles that were not freely available may have led to the omission of relevant studies accessible only through subscriptions or payment of fees.

### Contributions to the Nursing Field

In light of the above, this study constitutes a significant contribution to evidence-based practice, enhancing the decision-making capacity of nursing professionals operating in PHC. When addressing cases of DR-TB within this realm of care, nurses can now rely on clear and evidence-based guidance, thereby facilitating the delivery of comprehensive and coordinated care. However, it is crucial to underscore that the effectiveness of this approach hinges on the mastery of knowledge by professionals at all healthcare levels. It is imperative for these professionals to remain well-informed and updated, supported by robust ongoing health education, enabling them to address the diverse health needs of patients. Moreover, effective integration across different levels of care, facilitated by a well-established referral and counter-referral process, is essential to ensure comprehensive, continuous, and equitable care.

## FINAL CONSIDERATIONS

Amidst the current scenario of rising DR-TB cases, this study provides scientific evidence regarding the care that nurses should deliver in PHC, aimed at achieving care resolution. It underscores the necessity of policies to decentralize PHC-sensitive care services, transitioning them from specialized secondary and tertiary levels to the primary level, closer to individuals, families, and communities. Through collaboration between the three levels of healthcare, comprehensive and decisive care can be effectively achieved.

Therefore, this study highlights several aspects of care that should be provided by PHC nurses to individuals with DR-TB, including nursing care management for person-centered care, comprehensive nursing consultations utilizing the nursing process, integration of TB/HIV investigation and care, home visits, health education, Directly Observed Therapy (DOT), investigation of respiratory symptoms, and Latent Tuberculosis Infection (LTBI) treatment. To accomplish this, greater involvement and support for these professionals through ongoing health education are necessary to alleviate the fear induced not only by the disease but also by the sudden changes in daily practices.
