# Supplementary material for: Drug-resistant tuberculosis: integrative review of nursing care in primary health care
Source: Rev Bras Enferm. 2024 Jul 19;77(2):e20230097. doi: 10.1590/0034-7167-2023-0097 (PMC11259442; doi:10.1590/0034-7167-2023-0097)
Supplement: Translation

# Translation: Tuberculose drogarresistente: revisão integrativa dos cuidados de enfermagem na atenção primária à saúde

**DOI:** 10.1590/0034-7167-2023-0097pt

**Authors:** Sibele Naiara Ferreira Germano, Alacoque Lorenzini Erdmann, Camila Freire Albuquerque, Lúcia Nazareth Amante, Darlisom Sousa Ferreira, Marlucia da Silva Garrido

**Affiliations:** I Universidade Federal de Santa Catarina. Florianópolis, Santa Catarina, Brasil; II Universidade do Estado do Amazonas. Manaus, Amazonas, Brasil; III Universidade Federal do Amazonas. Manaus, Amazonas, Brasil

**Keywords:** Tuberculose, Tuberculose Resistente a Múltiplos Medicamentos, Enfermeiras e Enfermeiros, Cuidados de Enfermagem, Atenção Primaria à, Saúde

## Abstract

**Objetivos:**

identificar, na literatura científica, os cuidados que devem ser prestados às pessoas com tuberculose drogarresistente pelos enfermeiros na atenção primária à saúde.

**Métodos:**

revisão integrativa, utilizando o fluxograma de seleção de estudos *Preferred Reporting Items for Systematic Reviews and Meta-Analyses*. A coleta de dados foi realizada em novembro de 2022, em dez bases de dados.

**Resultados:**

seis estudos enfatizaram que o enfermeiro deve realizar o tratamento diretamente observado; dois trouxeram a importância da gestão do cuidado integrado entre tuberculose e vírus da imunodeficiência humana; dois evidenciaram a consulta de enfermagem abrangente com utilização do processo de enfermagem; um salientou os cuidados centrados na pessoa, com planejamento de alta e melhor comunicação hospitalar com os serviços de atenção primária à saúde.

**Considerações Finais:**

os cuidados que devem ser prestados pelos enfermeiros às pessoas com tuberculose drogarresistente na atenção primária à saúde, para resolutividade do cuidado, são baseados em evidências científicas.

## INTRODUÇÃO

Acreditava-se que a Tuberculose (TB) poderia ser eliminada até 2035 com o provimento de estratégias de controle, fornecimento de tratamento gratuito e avanços relacionados aos meios de diagnóstico precoce. Entretanto, o surgimento da pandemia de COVID-19 retardou os avanços na eliminação da TB como problema de saúde pública global, devido à redução de 25% no diagnóstico e aumento de 26% da mortalidade pela doença^(1-2)^. Isso se agrava ainda mais com o aumento da sua forma grave, a Tuberculose Drogarresistente (TB-DR), que teve uma estimativa recente de 450 mil novos casos segundo a Organização Mundial de Saúde (OMS)^(1)^.

A TB é definida como TB-DR quando o *Mycobacterium tuberculosis* torna-se resistente a uma ou mais drogas antituberculose. Existem cinco formas desta doença: a TB resistente a somente um medicamento (monorresistência); a resistente a dois ou mais medicamentos (polirresistência), exceto a associação rifampicina e isoniazida; a resistente detectada pelo teste rápido molecular para tuberculose (resistência à rifampicina); a resistente a, pelo menos, rifampicina e isoniazida (multirresistência); e a resistente à rifampicina e isoniazida, acrescida de resistência a qualquer fluoroquinolona e, pelo menos, um dos medicamentos bedaquilina e linezolida (extensiva resistência)^(2-3)^.

Dependendo da forma de TB-DR, determina-se o esquema de tratamento e o período, que pode variar de 18 a 24 meses ou mais, considerando a evolução clínica, radiológica e bacteriológica. A complexidade desse tratamento é maior por causa da necessidade de medicamentos de segunda linha, orais e/ou injetáveis, os quais podem produzir efeitos colaterais mais graves. Recentemente foram introduzidos novos esquemas de tratamentos totalmente orais para TB-DR, com a possibilidade de redução do tempo de tratamento em até 6 meses, comparado aos esquemas anteriores^(4)^.

Diante desse novo contexto, a OMS recomenda a expansão do acesso ao tratamento totalmente oral, reforçando a necessidade de fortalecimento dos cuidados de enfermagem na APS, que atua em nível local próximo da pessoa, família e comunidade, para apoio, aconselhamento e monitoramento de eventos adversos^(1,4)^. Destacando que os cuidados de enfermagem às pessoas com TB-DR precisam ser realizados de forma integral nos três níveis de atenção, primário, secundário e terciário; com a APS como coordenadora desse cuidado, porém esta precisa estar preparada com profissionais qualificados, dentre eles os enfermeiros de práticas avançadas que são essenciais para aumentar o acesso e fornecer melhor manejo às pessoas com essa doença neste nível de atenção^(5-6)^.

É notório que na APS a taxa de perda de seguimento de pessoas com TB-DR que recebem os cuidados de enfermagem é menor do que aqueles que só recebem cuidados hospitalares, e a taxa de sucesso do tratamento nas pessoas que recebem os cuidados dessa doença na APS é maior, comparados àqueles que só recebem cuidados no hospital^(7-8)^.

O Ministério da Saúde (MS) no Brasil, assim como outros países, reconhece o papel do enfermeiro de práticas avançadas nos cuidados às pessoas com TB-DR na APS e lançou em 2022 o protocolo de tratamento da TB para os enfermeiros com recomendações relacionadas à assistência à TB-DR na APS, onde consta a realização da consulta de enfermagem, o planejamento, gerenciamento, coordenação e avaliação das ações desenvolvidas por enfermeiros qualificados com competência clínica^(6,9-10)^.

Frente a este cenário de aumento da TB-DR, novos esquemas de tratamento oral e a necessidade de fortalecimento dos cuidados na APS, justifica-se este estudo pelo entendimento de que ao buscar na literatura os cuidados prestados por enfermeiros em pessoas com TB-DR na APS, será fornecido evidências científicas dos cuidados que devem ser realizados por esses profissionais ao lidarem com essa grave doença a nível local, com a participação da pessoa, família e comunidade, aumentando o acesso e a adesão ao tratamento correto até o alcance da cura desta doença que afeta a saúde pública global^(4-6)^.

## OBJETIVOS

Identificar, na literatura científica, os cuidados que devem ser prestados às pessoas com tuberculose drogarresistente pelos enfermeiros na atenção primária à saúde.

## MÉTODOS

Estudo descritivo de revisão integrativa, o qual sintetiza resultados de pesquisas científicas sobre um tema específico de maneira sistemática e ordenada, realizado em seis etapas: identificação do tema e definição da pergunta norteadora da pesquisa; estabelecimento de critérios para inclusão e exclusão dos estudos; categorização dos estudos; avaliação crítica dos estudos incluídos; interpretação dos resultados; descrição da síntese do conhecimento científico do tema investigado, contribuindo para a realização de práticas baseadas em evidências^(11)^.

A primeira etapa, definição da pergunta de pesquisa, surgiu da estratégia PICo, que significa: P- Participantes, I- Fenômeno de Interesse e Co- Contexto^(12)^. Neste estudo, atribuiu-se para P- enfermeiros, I- cuidados prestados às pessoas com tuberculose drogarresistente; Coatenção primária à saúde, resultando na seguinte pergunta norteadora: Quais os cuidados que os enfermeiros da atenção primária à saúde devem prestar às pessoas com tuberculose drogarresistente?

Os critérios de inclusão definidos para a busca dos estudos nas bases de dados foram: estudos qualitativos e/ou quantitativos; randomizados; que tratassem dos cuidados que devem ser realizados por enfermeiros em pessoas com tuberculose drogarresistente; utilizaram o critério de prática avançada de enfermagem no cuidado às pessoas com tuberculose drogarresistente na atenção primária; e cuidados realizados às pessoas com tuberculose drogarresistente pela equipe multiprofissional da atenção primária, constando o profissional enfermeiro; no contexto nacional e internacional; nos idiomas português, inglês e espanhol, publicados na íntegra nos últimos cinco anos (2018 a 2022). Os critérios de exclusão foram revisões de literatura, estudos reflexivos, relatos de experiência e os que não possuíam aderência à questão norteadora da pesquisa.

A coleta de dados foi realizada em novembro de 2022, nas bases de dados: *Cumulative Index to Nursing and Allied Health Literature (CINAHL),* Literatura Latino-Americana de Ciências da Saúde (LILACS), Banco de Dados em Enfermagem (BDENF)*, National Library of Medicine (PubMed)/ Medical Literature Analysis and Retrieval System Online (MEDLINE), Excerpta Medical Database (EMBASE), Cochrane Library, Scopus, Web of Science, Scientific Electronic Library Online (SciELO)*. As estratégias de busca foram adaptadas, com ajuda de uma bibliotecária, momento em que foi testado mais de uma estratégia para então chegar à definição final, utilizando as terminologias definidas pelo *Medical Subject Headings (MeSH)* e os descritores em Ciência da Saúde (DeCS) conforme as bases de dados, com utilização dos operadores booleanos *OR* e *AND*, descritas no Quadro 1.

**Quadro 1:** Bases de dados com estratégias de busca utilizando as terminologias *Medical Subject Headings* e Descritores em Ciência da Saúde, Florianópolis, Santa Catarina, Brasil, 2022

Base de dados	Estratégias de busca
*Cumulative Index to Nursing and Allied Health Literature* *National Library of Medicine/ Medical Literature Analysis and Retrieval System Online* *Excerpta Medical Database* *Cochrane Library* *Scopus* *Web of Science*	*(Tuberculosis OR “Koch Disease” OR “Koch’s Disease” OR “Kochs Disease” OR “Mycobacterium tuberculosis Infection” OR “Mycobacterium tuberculosis Infections” OR Tuberculoses OR “Tuberculosis, Multidrug-Resistant” OR “Drug-Resistant Tuberculosis” OR “MDR Tuberculosis” OR “Multi-Drug Resistant Tuberculosis” OR “Multidrug-Resistant Tuberculosis” OR “Tuberculosis, Drug Resistant”) AND (Nurses OR Nurse OR “Nursing Personnel” OR “Registered Nurse” OR “Registered Nurses” OR “Nursing Care” OR “Nursing Care Management”) AND (“Primary Health Care” OR “Health Care, Primary” OR “Primary Care” OR “Primary Healthcare”)*
Literatura Latino-Americana de Ciências da Saúde Banco de Dados em Enfermagem	(Tuberculose *OR* Tb *OR* “Infecção por *Mycobacterium tuberculosis*” *OR* “Pneumologia Sanitária” *OR* Tb *OR Tuberculosis OR “Infección por Mycobacterium tuberculosis”) AND* (“Enfermeiras e Enfermeiros” *OR* Enfermeira *OR* “Enfermeira e Enfermeiro” *OR* “Enfermeira Registrada” *OR* Enfermeiras *OR* “Enfermeiras Registradas” *OR* “Enfermeiro e Enfermeira” *OR* “Enfermeiro Registrado” *OR* “Enfermeiros e Enfermeiras” *OR* “Enfermeiros Registrados” *OR “Enfermeras y Enfermeros” OR Enfermera OR “Enfermera Registrada” OR “Enfermera y Enfermero” OR Enfermeras OR “Enfermeras Registradas” OR “Enfermero Registrado” OR “Enfermero y Enfermera” OR “Enfermeros Registrados” OR “Enfermeros y Enfermeras” OR* “Cuidados de Enfermagem” *OR* “Assistência de Enfermagem” *OR* “Atendimento de Enfermagem” *OR* “Cuidado de Enfermagem” *OR* “Gestão da Assistência de Enfermagem” *OR* “Sistematização da Assistência de Enfermagem” *OR* “Atención de Enfermería” *OR* “Cuidado de Enfermería” *OR* “Cuidados de Enfermería”)
*Scientific Electronic Library Online*	*(“Tuberculose Resistente a Múltiplos Medicamentos” OR “Tuberculosis Resistente a Múltiples Medicamentos”) AND (Enfermeiros OR Enfermeros OR “Cuidados de enfermagem” OR “Atención de Enfermería” OR Enfermagem)*

O acesso às bases de dados ocorreu por meio do Portal de Periódicos da Coordenação de Aperfeiçoamento de Pessoal de Nível Superior em área com *Internet Protocolo* (IP) reconhecida da Universidade Federal de Santa Catarina, e a busca, seleção e inclusão dos estudos foram realizadas por dois revisores de forma independente, usando o fluxograma de seleção de estudos *Preferred Reporting Items for Systematic Reviews and Meta-Analyses* (PRISMA)^(13)^. Em situações divergentes, buscou-se um consenso com o apoio do terceiro revisor, que emitiu um parecer para a decisão quanto à inclusão. As listas dos estudos selecionados nesta fase foram comparadas e verificou-se a concordância da análise através do teste de Kappa^(14)^.

O gerenciador bibliográfico *EndNote Basic* ^®(15)^, versão *web*, auxiliou na organização das buscas, excluindo os artigos duplicados. A exportação das publicações encontradas foi realizada pelo *Mendeley^®^ Data Manager* ^(16)^. Esses dados foram posteriormente agrupados e exportados em um arquivo RIS, utilizando o *software MS Office Excel* ^®^, para complementar o processamento de extração de dados.

Os dados extraídos foram organizados e sintetizados com a utilização de um formulário específico construído pelos pesquisadores, contendo as seguintes informações: identificação do(s) autor(es), título, periódico, país, idioma do estudo e ano de publicação; objetivo; metodologia do estudo; cuidados que devem ser prestados às pessoas com TB-DR na APS, principais resultados, conclusões, limitações metodológicas (risco de viés), inconsistência, evidência indireta, imprecisão, viés de publicação e nível de evidência dos estudos pelo *Grading of Recommendations Assessment Developing Evaluation* (GRADE)^(17)^_._

As estratégias de busca permitiram recuperar 1.110 artigos nas bases de dados. Destes, 914 foram excluídos após a aplicação dos filtros, conforme os critérios pré-estabelecidos, resultando em 196 para leitura de títulos e resumos, dos quais foram excluídos 170. Foram elegíveis 26 estudos para leitura completa, restando 11 estudos que responderam à questão de pesquisa dessa revisão, conforme fluxograma da Figura 1.

**Figura 1:** Fluxograma de seleção de estudos adaptado do *Preferred Reporting Items for Systematic Reviews and Meta-Analyses* 2020^(13)^, Florianópolis, Santa Catarina, Brasil, 2022

## RESULTADOS

Os estudos incluídos na descrição e síntese desta revisão^(18-28)^ trazem como resultado os cuidados que devem ser prestados por enfermeiros na APS às pessoas com TB-DR, evidenciando a importância desses profissionais na promoção da saúde, prevenção e tratamento adequado à doença. Esses cuidados são embasados em protocolos e diretrizes que surgem de políticas de saúde pública abrangente, que enxergam a pessoa, a família e a comunidade no seu contexto de vida e dentro de um sistema de saúde com referência e contrarreferência, articulando os três níveis de atenção primário, secundário e terciário para o cuidado integral e resolutivo, conforme apresentado no Quadro 2.

**Quadro 2:** Identificação dos artigos publicados entre 2018-2022 com descrição dos cuidados de enfermagem às pessoas com Tuberculose Drogarresistente na Atenção Primária à Saúde, Florianópolis, Santa Catarina, Brasil, 2022

Título do estudo	Ano País	Delineamento metodológico	Principais resultados/conclusões dos cuidados de enfermagem	Nível de evidência GRADE
*An intervention to optimise the delivery of integrated tuberculosis and HIV services at primary care clinics: results of the MERGE cluster randomised trial^(18)^*	2018 África do Sul	Estudo Randomizado, em cluster.	Gestão do cuidado integrado para TB/HIV com diagnósticos precoces e tratamento conforme diretriz, com rastreio dos contatos.	Alta
*Nurses’ Knowledge of Tuberculosis, HIV, and Integrated HIV/TB Care Policies in Rural Western Cape, South Africa^(19)^*	2018 África do Sul	Qualitativo e quantitativo	Gestão do cuidado integrado TB/HIV após extensivo treinamento dos profissionais nas diretrizes de TB e em sua forma grave TB-DR.	Alta
*Continuity of care for TB patients at a South African hospital: A qualitative participatory study of the experiences of hospital staff^(20)^*	2019 África do Sul	Pesquisa-ação	Cuidados centrados na pessoa, com planejamento de alta e melhor comunicação do hospital com os serviços de Atenção Primária à Saúde.	Baixa
Percepções de enfermeiros sobre gestão do cuidado e seus fatores intervenientes para o controle da tuberculose^(21)^	2022 Brasil	Qualitativo	Consulta de enfermagem, monitoramento dos casos confirmados com baciloscopias de controle; realização do TDO; realização do tratamento da ILTB; e realização de educação em saúde.	Moderada
Consulta de enfermagem a pessoas com TB: proposta de instrumento^(22)^	2020 Brasil	Pesquisa-ação	Consulta de enfermagem às pessoas com TB e TB-DR utilizando o Processo de Enfermagem, contemplando todas as etapas, promovendo cuidado individualizado e adequado para cada pessoa.	Moderada
*Representations on adherence to the treatment of Multidrug-Resistant Tuberculosis^(23)^*	2018 Brasil	Qualitativo	Acolhimento, escuta, atenção, esclarecimento de dúvidas e criação de vínculo. Realização do TDO para adesão ao tratamento e acompanhamento das reações adversas à medicação; educação em saúde para a pessoa doente e familiares.	Baixa
*Coordination and list of services for the management of tuberculosis: perspective of health professionals^(24)^*	2022 Brasil	Quantitativo	TDO com visitas domiciliares para acompanhamento e controle mensal dos doentes e seus contatos; realização do teste rápido de HIV em todas as pessoas com TB; garantia de fluxo com os serviços de referência e contrarreferência em TB, TB-DR e HIV.	Baixa
*Nurses’ discourses on actions and strategies of care for the elderly with tuberculosis^(25)^*	2022 Brasil	Qualitativo	Cuidado integral com busca ativa, incentivos, visitas domiciliares, educação em saúde e exames.	Muito Baixa
Percepção de enfermeiros: enfoque na família e orientação para a comunidade nas ações de TB^(26)^	2020 Brasil	Quantitativo	Inclusão da pessoa com TB na elaboração do plano de cuidados e a sua participação no planejamento, o respeito e incentivo à autonomia para realizar o TDO, com fortalecimento de vínculo, desenvolvimento de ações de promoção e prevenção, rastreamento dos sintomáticos respiratórios e educação em saúde.	Muito Baixa
Passos e descompassos no processo de cuidado aos portadores de tuberculose na atenção primária^(27)^	2020 Brasil	Qualitativo	Cuidado conforme políticas e estratégias da Organização Mundial da Saúde: diagnóstico precoce com busca ativa de SR, testagem para HIV, TDO e educação permanente instigando sempre a prática baseada em evidências.	Baixa
*Provider perspectives of the introduction and implementation of care for drug-resistant tuberculosis patients in districtlevel facilities in South Africa: a qualitative study^(28)^*	2020 África do Sul	Qualitativo	Cuidados descentralizados com realização do TDO, monitoramento de efeitos colaterais e adesão por meio de escarro mensal e testes de rotina.	Moderada

*TB - Tuberculose; TB-DR* - *Tuberculose Drogarresistente; HIV - Vírus da Imunodeficiência Humana; TDO - Tratamento Diretamente Observado; ILTB - Infecção Latente pelo Mycobacterium Tuberculosis; SR - Sintomático Respiratório.*

Com relação aos cuidados de enfermagem que devem ser realizados pela APS, seis estudos^(21,24,26-28)^ enfatizam o Tratamento Diretamente Observado (TDO), dois^(18-19)^ trazem a importância da gestão do cuidado integrado TB/HIV, dois^(21-22)^ evidenciam a consulta de enfermagem abrangente com utilização do Processo de Enfermagem (PE), e um^(18)^ destaca os cuidados centrados na pessoa doente com planejamento de alta e melhor comunicação hospital com os serviços de APS.

Além desses cuidados, os estudos^(18-28)^ citam outros cuidados, que também estão presentes nas políticas e estratégias da OMS, como acolhimento, escuta, atenção, esclarecimento de dúvidas, criação de vínculo, diagnóstico precoce, visita domiciliar com busca ativa de Sintomáticos respiratórios (SR), realização do teste rápido de HIV em todas as pessoas com TB com garantia de fluxo com os serviços de referência e contrarreferência em TB, TB-DR e HIV, realização do tratamento da ILTB, educação permanente e em saúde, instigando sempre a prática baseada em evidências.

Ao verificar as características dos estudos elencados e seus níveis de evidência, sete estudos^(21-27)^ foram realizados no Brasil e quatro^(18-20,28)^ na África do Sul. Cinco desses estudos^(21,23,25,27-28)^ apresentaram delineamento metodológico qualitativo, dois^(22,24)^ quantitativo, dois pesquisa-ação^(18,20)^_,_ um^(19)^ qualitativo e quantitativo e um^(18)^ randomizado em cluster.

Ao avaliar os fatores que diminuem a confiança nos resultados (vieses dos estudos, inconsistência, imprecisão, evidência indireta e viés de publicação) e os fatores que aumentam (tamanho grande de efeito, gradiente dose resposta, vieses conservadores de cada estudo seguindo o sistema GRADE)^(17)^, dois artigos^(18-19)^ contemplaram os itens necessários para serem considerados de alta qualidade metodológica e nove^(20-28)^ não conseguiram atingir esse nível de evidência, devido à ausência ou insuficiência da descrição metodológica dos itens necessários.

## DISCUSSÃO

Diante da importância do manejo e cuidado adequado da TB para o controle da TB-DR, é essencial considerar que pessoas com TB previamente tratadas são mais propensas a desenvolver a TB-DR, seja devido a esquemas de tratamento inadequados ou à não adesão à terapêutica^(2-3)^. Além disso, as pessoas com TB-DR representam uma fonte de disseminação de cepas resistentes a outros indivíduos que podem adquirir essa forma grave da doença primariamente^(10)^.

Nesse contexto e considerando a necessidade de um cuidado mais específico às pessoas com TB-DR, destacamos em dois tópicos os cuidados de enfermagem que devem ser prestados a essas pessoas na APS, conforme emergiram dos estudos analisados.

### Continuidade dos cuidados de enfermagem em pessoas com tuberculose drogarresistente na atenção primária à saúde

Estudos realizados com pessoas com TB-DR, utilizando a continuidade do cuidado entre o hospital, centro de referência e a APS, demonstraram que essa abordagem foi eficiente para o tratamento dessas pessoas, especialmente quando há a presença de enfermeiros de prática avançada no cuidado no nível primário. Essa modalidade de cuidado próximo da pessoa doente evidencia um aumento do acesso aos serviços de saúde, um melhor manejo da doença e uma maior satisfação dos usuários^(23,28)^. Esses resultados foram observados não apenas em estudos realizados no Brasil, mas também em estudos conduzidos na África do Sul e no Peru, onde o atendimento em clínicas próximas às residências das pessoas com TB-DR ou atendimento domiciliar foram alternativas eficazes à hospitalização durante todo o tratamento^(29-30)^.

Os cuidados de enfermeiros em pessoas com TB-DR devem ser realizados com continuidade nos três níveis de atenção (primário, secundário e terciário), visando o cuidado integral e constante vigilância à TB-DR^(4,10,23,28)^. Estudos demonstram que o enfermeiro da APS deve realizar consulta de enfermagem às pessoas com TB-DR, solicitar exames complementares, executar o Tratamento Diretamente Observado (TDO), orientar sobre reações adversas à medicação conforme protocolos clínicos e diretrizes terapêuticas, além de supervisionar o acolhimento com escuta qualificada e classificação de risco. Também é papel do enfermeiro elaborar planos de cuidados, coordenar e avaliar as ações da equipe, incluindo técnicos/auxiliares de enfermagem e agentes comunitários de saúde (ACS), orientando a busca de sintomáticos respiratórios na área (SR)^(6,10,23,28)^.

Todos os cuidados realizados pelos enfermeiros devem seguir as diretrizes do Gerenciamento Programático da Tuberculose Resistente a Medicamentos (GPTRM), formulado pela OMS. Vários países, como a África do Sul, Peru e Brasil, possuem protocolos clínicos e diretrizes terapêuticas específicos para os enfermeiros da APS, fortalecendo esse nível de atenção ao descrever os cuidados que os enfermeiros devem realizar frente às pessoas com TB-DR, incluindo a avaliação clínica por meio do exame físico durante a consulta de enfermagem, a execução do TDO com avaliação dos comunicantes para infecção latente da TB (ILTB)^(6,29-30)^.

É evidente a necessidade dos cuidados dos enfermeiros em pessoas com TB-DR na APS. Um estudo realizado em um hospital de referência em casos graves de TB mostrou falhas no cuidado das pessoas doentes que tinham apenas seu tratamento realizado pela atenção terciária (hospitalar). Os enfermeiros expressaram preocupação com a não realização do TDO, a falta de seguimento dos cuidados às pessoas com TB-DR e o acompanhamento dos familiares, que deveriam ser realizados neste nível de atenção, sem a devida referência e contrarreferência e sem o envolvimento da equipe de saúde da APS no plano de alta e continuidade do cuidado^(20)^.

Estudos mostram que essa falta de continuidade do cuidado do nível especializado para a atenção primária à saúde propicia falhas no cuidado integral, pela falta de atenção à saúde das pessoas com TB-DR, de sua família e comunidade, excluindo-os do planejamento de alta. Isso resulta na ausência de continuidade dos cuidados sensíveis à APS do nível central para o local, deixando o enfermeiro da APS incapaz de realizar na consulta de enfermagem o TDO, a educação e aconselhamento das pessoas com a doença e familiares, prejudicando esse cuidado e aumentando os casos de abandono de tratamento e disseminação da doença^(20,23)^.

Descobertas semelhantes foram encontradas em estudos realizados em países com alta carga de TB que não possuíam integração efetiva do cuidado entre hospitais e serviços de TB na APS. Estratégias tiveram que ser implementadas para integrar os cuidados realizados nos hospitais gerais e serviços de APS no Programa Nacional de Controle da TB e sua forma grave, a TB-DR. Destaca-se a importância das estruturas de coordenação entre os níveis, sendo a APS considerada um elo na organização do cuidado entre os níveis de atenção, por estar mais próxima das pessoas. Os profissionais devem realizar a prevenção e a detecção precoce da doença, encaminhando para atenção especializada sempre que necessário, com compartilhamento das informações^(31-33)^.

Todavia, um estudo destacou a importância das pessoas com TB-DR iniciarem seu tratamento em unidades de referência em TB-DR, e posteriormente serem encaminhadas para APS, onde os enfermeiros devem realizar as injeções diárias, executar o TDO e monitorar o exame de escarro. Mensalmente ou bimestralmente, essas pessoas doentes devem retornar à unidade de referência para revisão do tratamento com base nos resultados do monitoramento mensal do exame de escarro na APS^(28)^.

É evidente em vários estudos^(7-9,20,23,28)^ que o cuidado realizado na APS em pessoas com TB-DR é uma realidade bem-sucedida, utilizada em alguns países, que propõe o tratamento ambulatorial, com enfermeiros acompanhando os cuidados, propiciando boas condições e redução do tempo de internação para aqueles que necessitam de internação. O compartilhamento de responsabilidade pelo cuidado e tratamento adequado nos três níveis de atenção, com a descentralização do nível central para o distrital e local, para a realização de um cuidado de enfermagem mais próximo, centrado na pessoa, melhora sua adesão e o custo-efetividade do tratamento.

### Cuidados de enfermagem centrados na pessoa com tuberculose drogarresistente, família e comunidade.

Diante do aumento dos casos de TB-DR, destaca-se a necessidade dos cuidados de enfermagem centrados na pessoa para o controle dessa doença. Os profissionais enfermeiros da APS desempenham um papel crucial desde a busca ativa de sintomáticos respiratórios até a concretização do diagnóstico, realização e acompanhamento do tratamento. Eles aplicam o Tratamento Diretamente Observado (TDO), monitoram os casos confirmados com baciloscopias de controle até o final do tratamento e controlam diariamente os faltosos, utilizando estratégias para evitar o abandono, como a Visita Domiciliar (VD) e a educação em saúde^(21-22,34)^.

Estudos comprovam que as visitas domiciliares e as atividades de educação em saúde aumentam o rastreio dos contatos, aproximam a família do profissional e reduzem o estigma da doença^(14,18,20-23)^. Essas atividades são fundamentais para o enfrentamento da TB, influenciando a adoção de medidas relevantes à terapêutica, como o TDO, para a continuidade da tomada da medicação e manutenção do acompanhamento na APS^(19,26)^.

O TDO é uma estratégia de cuidado lançada pela OMS para fortalecer a capacidade de resposta contra a TB. Seus principais objetivos incidem na adesão das pessoas ao tratamento padronizado com supervisão da tomada da medicação e apoio social, prevenção do aparecimento de cepas resistentes aos medicamentos, redução dos casos de abandono e aumento da probabilidade de cura da doença^(23,27)^.

Os enfermeiros da APS, ao realizarem o cuidado centrado na pessoa com TB-DR, atuam também no controle de contatos, tratamento quando indicado da infecção latente da TB (ILTB), preenchimento dos instrumentos de vigilância preconizados pelo Programa Nacional de Controle da Tuberculose (PNCT) e realização de atividades de educação em saúde, enfatizando a promoção à saúde e prevenção da doença^(21-22,24,34)^.

Estudos comprovam que o enfermeiro, ao realizar a Gestão do Cuidado de Enfermagem (GCE), fornece um cuidado integral e efetivo após receber extensa educação permanente em saúde (EPS) sobre TB e TB-DR^(19-20)^. Isso demonstra a necessidade de permanente atualização do conhecimento dos enfermeiros sobre diagnóstico, tratamento e acompanhamento da TB-DR, com necessidade de um sistema de apoio para que o tratamento seja bem-sucedido, bem como a adoção das políticas, protocolos e sistemas eletrônicos de informação para registro, notificação e compartilhamento de informações entre o hospital e os serviços de APS.

Os cuidados de enfermagem centrados na pessoa com TB-DR na APS refletem atualmente uma prática que precisa ser fortalecida por meio da EPS. As iniquidades ainda estão presentes entre os profissionais em relação aos níveis instrucionais no Brasil, exigindo um maior protagonismo desses sujeitos frente às atitudes e tomada de decisão na APS para prevenção dessa doença e promoção de saúde, executando o que é ideal, em oposição ao real, instigando sempre a prática baseada em evidências^(27)^.

Estudos evidenciam que para desenvolver o cuidado centrado na pessoa, deve-se realizar a Gestão do Cuidado de Enfermagem (GCE) integral, onde o enfermeiro utiliza ferramentas de gestão, como indicadores de saúde, planejamento de materiais e recursos humanos, normas de segurança para atendimento e processo de tomada de decisão^(19-21)^. Para isso, o cuidado prestado diretamente ao público deve englobar técnicas, tecnologias, procedimentos e ações de prevenção, promoção e educação em saúde^(21,35)^.

Destaca-se que os profissionais que atuam realizando a GCE das pessoas com TB-DR precisam ser preparados para o cuidado centrado na pessoa em todas as suas dimensões, de modo que compreendam seu papel e estejam capacitados para acompanhar esses usuários, demonstrando-lhes que podem e devem atuar em prol da própria saúde^(21,35)^.

Assim, os cuidados de enfermagem devem ser centrados na pessoa doente, fortalecendo a democracia e adesão ao tratamento correto, incluindo-os na elaboração do plano de cuidados e no planejamento, com respeito e incentivo à autonomia na realização do TDO. Envolve a participação da população nas discussões sobre o assunto e utilização de recursos comunitários pelos profissionais para apoiar a adesão a esse método^(26)^.

O protocolo de assistência à TB destaca que, para garantir o cuidado de enfermagem integral centrado na pessoa doente, é necessário que os enfermeiros realizem a consulta de enfermagem de forma ampla, utilizando as etapas do Processo de Enfermagem: investigação, diagnóstico, planejamento, implementação e avaliação, promovendo cuidado individualizado e adequado para cada pessoa, subsidiado por referenciais teóricos^(21-22,35)^.

Ao iniciar a etapa de investigação das pessoas com TB-DR, alguns aspectos devem ser contemplados no exame físico, devendo-se investigar os sinais e sintomas da doença, há quanto tempo se apresenta, contatos com indivíduo com tuberculose, exames realizados durante o tratamento, acompanhamento profissional, assistência de enfermagem, percepções e preocupações quanto à doença e realização de baciloscopia de acompanhamento^(23)^.

Durante o exame físico dessas pessoas com TB-DR, o enfermeiro deve estar atento para taquicardia, taquipneia ou dispneia de esforço, frequência respiratória aumentada, sons respiratórios diminuídos ou ausentes bilateral ou unilateralmente, estertores pós-tussígenos, assimetria na excursão respiratória (em casos de derrame pleural), macicez à percussão e diminuição do frêmito (em casos de haver líquido pleural). Observar também estertores crepitantes finos após a tosse, murmúrios vesiculares diminuídos ou com sopros anfóricos. Pode estar presente dor torácica agravada com tosse recorrente. Doenças associadas como diabetes e HIV devem ser pesquisadas^(21-22,36)^.

Um dos estudos^(18)^ realizado em 18 clínicas de APS demonstrou que as pessoas com TB necessitam ser investigadas imediatamente para sua principal coinfeção, o HIV, para reduzir a morbimortalidade pela coinfeção TB/HIV, com realização de aconselhamento e testagem rápida para HIV em todas as pessoas com TB, intensificando a busca ativa de casos de TB em casos de HIV e vice-versa, terapia preventiva, para controle de infecção por TB e início precoce da Terapia Antirretroviral (TARV).

O Ministério da Saúde do Brasil, em consonância com a OMS, recomenda que todo indivíduo com diagnóstico de TB deve ser testado para HIV, uma vez que o diagnóstico precoce possui importante impacto no curso clínico de ambas as doenças. Os casos positivos devem ser encaminhados para o Serviço de Atenção Especializada (SAE) ou unidades dispensadoras de medicamentos, a fim de dar continuidade ao tratamento da TB e iniciar o de HIV, realizando sempre a referência e contrarreferência dentro da Rede de Atenção à Saúde (RAS)^(24)^. Portanto, é evidente a necessidade de toda pessoa com TB-DR ser atendida de forma integral, conforme suas necessidades e comorbidades, por uma equipe multiprofissional na qual o enfermeiro da APS atua como membro da equipe além de gestor e coordenador do cuidado^(18-29,35)^.

### Limitações do estudo

As limitações deste estudo incluem predominantemente a abordagem qualitativa dos dados. Embora essa abordagem envolva amostras significativas e clinicamente relevantes, sua natureza qualitativa não permite estabelecer relações de causa e efeito. Isso ocorre porque o envolvimento da pessoa doente em seu cuidado pode variar em diferentes cenários e grupos, dificultando a generalização dos resultados. Outra limitação é a não exploração da literatura cinzenta, que poderia fornecer insights adicionais e uma visão mais abrangente sobre o tema. Além disso, a exclusão de artigos que não estavam disponíveis gratuitamente pode ter resultado na perda de estudos relevantes que estavam acessíveis apenas por meio de assinaturas ou pagamento de taxas.

### Contribuições para a área da enfermagem

Diante do exposto, este estudo representa uma contribuição significativa para a prática baseada em evidências científicas, fortalecendo a capacidade de tomada de decisão dos profissionais de enfermagem que atuam na APS. Ao lidar com casos de Tuberculose Drogarresistente (TB-DR) nesta esfera de cuidados, os enfermeiros podem agora contar com orientações claras e embasadas, facilitando a prestação de um cuidado integral e coordenado. No entanto, é importante ressaltar que essa eficácia depende do domínio do conhecimento por parte dos profissionais em todos os níveis de atenção à saúde. É essencial que esses profissionais estejam bem informados e atualizados, com base em uma educação permanente em saúde sólida, para que possam atender às diversas necessidades de saúde dos pacientes. Além disso, a integração eficaz entre os diferentes níveis de atenção, por meio de um processo de referência e contrarreferência bem estabelecido, é fundamental para garantir um cuidado integral, contínuo e equitativo.

## CONSIDERAÇÕES FINAIS

Diante do atual cenário de aumento dos casos de TB-DR, este estudo apresenta evidências científicas dos cuidados que devem ser prestados pelos enfermeiros na Atenção Primária à Saúde, visando à resolubilidade do cuidado. Destaca-se a necessidade de políticas de descentralização desses cuidados sensíveis à APS, do nível especializado secundário e terciário para o primário, mais próximo da pessoa, família e comunidade, mediante uma cooperação entre os três níveis de atenção à saúde, para alcançar efetivamente o cuidado integral e resolutivo.

Assim, este estudo aponta vários cuidados que devem ser prestados pelos enfermeiros da APS às pessoas com TB-DR, como a gestão do cuidado de enfermagem para o cuidado centrado na pessoa doente, por meio da consulta de enfermagem abrangente com utilização do processo de enfermagem, integração da investigação e do cuidado TB/HIV, realização da visita domiciliar, educação em saúde, TDO, investigação dos sintomáticos respiratórios e tratamento da ILTB. Para isso, é necessário um maior envolvimento e apoio a esses profissionais por meio de EPS, para eliminar o medo evocado não apenas pela doença, mas também pela mudança repentina nas práticas diárias.
